# Metrics and evidence for healthy ageing

**DOI:** 10.2471/BLT.19.246801

**Published:** 2019-12-01

**Authors:** Ritu Sadana, Anshu Banerjee, on behalf of the WHO Consortium on Metrics and Evidence for Healthy Ageing

**Affiliations:** a Department of Maternal, Newborn, Child and Adolescent Health, and Aging, World Health Organization, avenue Appia 20, 1211 Geneva 27, Switzerland.

The World Health Organization’s (WHO’s) response to population ageing is to promote healthy ageing across the life-course. Healthy ageing is the process of developing and maintaining the functional ability that enables well-being in older age.^1^

Endorsed in 2016, WHO’s Global Strategy and Action Plan on Ageing and Health^1^ describes what governments, international agencies and other partners can do to meet the needs and aspirations of older individuals. The action plan calls for a transformation in policies and institutions to enable interlinked actions that embrace diversity and narrow health inequities.^2^ Multisectoral efforts that bring together stakeholders from public, private, civil society and non-profit sectors,^3^ including older adults, are needed to adapt environments, so people can be and do what they value.

A second action plan on ageing and health, a Decade of Healthy Ageing (2020–2030), is under preparation. In 2020, WHO will also issue a global baseline report. The report will quantify the baseline indicators of healthy ageing; provide projections of Member State-endorsed outcome and impact indicators through 2030; and suggest ways to increase impact between 2020 and 2030 through illustrative, evidence-informed interventions that would enable countries to improve older adults’ intrinsic capacities and functional ability.

In 2017, WHO launched the International Consortium on Metrics and Evidence for Healthy Ageing consisting of policy-makers, members of civil society and researchers from all WHO regions. This consortium conducts research and evidence synthesis on healthy ageing, with work progressing to inform the baseline report. Here, we argue that this work and other efforts should promote inclusive dialogue between policy and scientific evidence for several reasons. First, the perspective and priorities of policy-makers in ministries and national institutes can guide the provision of evidence. Eight case studies from Chile, China, Finland, Ghana, India, Qatar, Singapore and Thailand, representing all WHO regions, are currently examining how evidence is being used by stakeholders to inform policy, make programmatic decisions and identify interventions that can improve the lives of older persons. These national case studies will identify common and distinct challenges, highlighting the importance of context-specific variation that can influence how policies are developed and what contributes to impact. For example, community-based organizations and social innovations can bridge health and social services at home and in communities,^4^ and also disseminate and implement interventions.^5^ Initiatives engaging older adults as contributors, not only recipients, can empower older people and promote social cohesion.

Second, tracking progress on healthy ageing over the coming decade should reflect collective benchmarking.^6^ Data and knowledge should be generated and shared jointly to help evaluating progress towards a common goal. Clarifying and testing standardized ways to operationalize and measure healthy ageing will lead to meaningful and relevant information. WHO’s *International classification of functioning, disability and health*,^7^ the only person-centred normative classification system of health that also recognizes context matters, provides a common basis for operationalization. Research to validate the proposed components of healthy ageing and measures should engage stakeholders and draw on the best available data sources, whether collected through population-based studies, geographic information systems or mobile devices. For example, nationally representative studies inclusive of older adults provide a wealth of data to analyse the structure of healthy ageing components in diverse populations and dynamics over time.^8^^–^^11^

Finally, evidence-informed decisions are required to increase impact. Evidence syntheses can be contributions to policy, guidance and research priorities. Ways to scale up promising pilot projects, ensure cost–effectiveness in diverse settings and reduce research waste, are further areas for improvement. Clinical trial evidence for chronic disease management is important; however, research on person-centred outcomes should be prioritized, using outcomes that older persons find meaningful, such as meeting basic needs, learning and making decisions, mobility, building and maintaining relationships, and contributing to families, communities or society – all of which are components of functional ability.^12^ Promoting these outcomes will also influence the design of future research, including innovations from low- and middle-income countries. These efforts will have the potential to improve peoples’ lives during the Decade of Healthy Ageing and beyond.
